# Integrating Machine Learning and Multi-Omics to Explore Neutrophil Heterogeneity

**DOI:** 10.3390/biomedicines13092171

**Published:** 2025-09-05

**Authors:** Zhiqiang Lin, Tingting Yang, Deng Chen, Peidong Zhang, Jialiu Luo, Shunyao Chen, Shuaipeng Gu, Youxie Shen, Tingxuan Tang, Teding Chang, Liming Dong, Cong Zhang, Zhaohui Tang

**Affiliations:** 1 Department of Trauma Surgery, Emergency Surgery and Surgical Critical, Tongji Trauma Center, Tongji Hospital, Tongji Medical College, Huazhong University of Science and Technology, Wuhan 430030, China; linzhiqiang@hust.edu.cn (Z.L.); chendeng@tjh.tjmu.edu.cn (D.C.); peidongzhang@hust.edu.cn (P.Z.); luojialiu@hust.edu.cn (J.L.); tjshunyaochen@hust.edu.cn (S.C.); gusp@hust.edu.cn (S.G.); shenyouxie@hust.edu.cn (Y.S.); changtd@tjh.tjmu.edu.cn (T.C.); lmdong@tjh.tjmu.edu.cn (L.D.); tangzh@tjh.tjmu.edu.cn (Z.T.); 2 Department of Radiology, Union Hospital, Tongji Medical College, Huazhong University of Science and Technology, Wuhan 430022, China; yangtt877@163.com; 3 Department of Orthopedics, Tongji Hospital, Tongji Medical College, Huazhong University of Science and Technology, Wuhan 430030, China; tangtingxuan@hust.edu.cn

**Keywords:** neutrophil heterogeneity, multi-omics, artificial intelligence, machine learning

## Abstract

Traditionally considered as homogeneous innate immune cells, neutrophils are now found to exhibit phenotypic and functional heterogeneity. How to determine whether the functional changes of neutrophils are caused by activation or the result of gene reprogramming? Recent advances in multi-omics technologies, including genomics, transcriptomics, proteomics, metabolomics, and spatial omics, have comprehensively explained the mechanism of neutrophil heterogeneity. At the same time, artificial intelligence, especially machine learning, has promoted the in-depth analysis of multi-omics. Here, we introduce the latest progress in the discovery of neutrophil subsets by omics research. We will further discuss the application of machine learning in analyzing the heterogeneity of neutrophils through omics methods. Our goal is to provide a comprehensive overview of how machine learning and multi-omics are reshaping our understanding of neutrophil biology and pathophysiology.

## 1. Introduction

Neutrophils, a type of innate immune cell, are the first responders to inflammation and play a crucial role in the body’s defense against pathogens [1]. These highly versatile cells mediate a range of functions, including degranulation, production of reactive oxygen species (ROS), phagocytosis, formation of neutrophil extracellular traps (NETs), and immune regulation [2,3]. Against the previous consensus of neutrophil homogeneity, neutrophils may remain in the circulation long enough to interpret environmental signals and execute specific molecular programs, which rationalize the neutrophil diversity in vivo [4,5,6]. Research into neutrophil subpopulations suggested a complex landscape of functional and phenotypic diversity. Influenced by environmental factors and pathogen signals, the presence of cytokines and chemokines can polarize neutrophils into distinct subpopulations [6]. However, traditional approaches to neutrophil heterogeneity research face limitations. Flow cytometry’s reliance on predefined markers limits the discovery of potential subpopulations and functional diversity [7]. Bulk sequencing obscures individual cellular variations through signal averaging [8], while single time-point sampling fails to capture dynamic phenotypic transitions during development and inflammation [9]. The mechanism of neutrophil heterogeneity is still a mystery. The defects of these methods hinder a comprehensive understanding of neutrophil plasticity, particularly the core characteristics of neutrophils, namely transcriptional and epigenetic properties [6]. Is heterogeneity a consequence of cellular programming or a basic functional activity of the cell? How to distinguish between a short activation phenomenon and a stable function reprogramming event? The limitations in neutrophil research methodology, particularly the lack of standardized metrics for assessing functional modulation, hinder precise characterization of the cellular states. The contradictions in findings from separate studies highlight the inadequacies of these methods in providing a holistic view.

To address the current limitations and advance our understanding, there is an urgent need for holistic and integrative approaches [10].

In response to the need for a comprehensive understanding of neutrophil heterogeneity, advances in multi-omics technologies, including genomics, transcriptomics, proteomics, metabolomics, and spatial omics, have begun to unravel the complex layers of neutrophil biology at an unprecedented resolution [11]. At the same time, the development of artificial intelligence (AI), especially machine learning (ML), assists in analyzing the massive data generated by these multi-level analyses. AI-driven analysis can uncover complex patterns in large datasets, while multi-omics can offer a holistic view of molecular mechanisms [12,13]. This synergy promises to indicate novel functional subsets of neutrophils and their roles in health and disease. Continual development in computational tools and data integration techniques will be essential to deeply realize their potential in neutrophil research and open new avenues for targeted therapies and personalized medicine.

In this review, following a brief introduction to the phenotypic and functional heterogeneity of neutrophils, we will first present recent advances in the discovery of neutrophil subsets through omics research. We will further explore the application of ML in unraveling neutrophil heterogeneity through omics approaches. We aim to provide a comprehensive overview of how ML and multi-omics reshape our understanding of neutrophil biology and pathophysiology.

## 2. Heterogeneity of Neutrophils in Phenotypes and Functions

**Figure 1:**
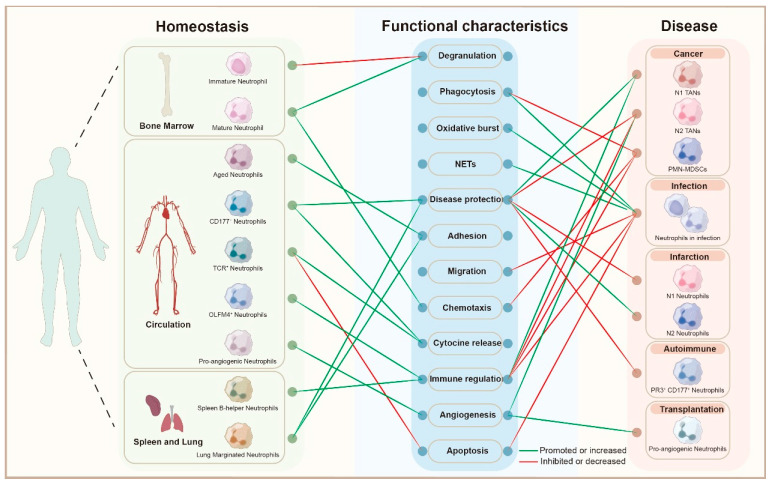
**Neutrophil Heterogeneity Across Homeostasis and Disease States:** this bipartite graph illustrates the functional characteristics of different neutrophil subsets across various stages of homeostasis and disease. On the left, neutrophil subpopulations derived from different anatomical sites (bone marrow, circulation, spleen, and lung) are depicted, including immature, mature, aged, and specialized subsets such as CD177^+^, TCR^+^, OLFM4^+^ neutrophils, and pro-angiogenic neutrophils. These subsets are linked to their functional characteristics, including degranulation, phagocytosis, oxidative burst, NET formation (NETs), disease protection, adhesion, migration, chemotaxis, cytokine release, immune regulation, angiogenesis, and apoptosis, as shown in the central node list. The graph contrasts the functional roles of neutrophils under homeostasis (green edges) and in various disease contexts (red edges). The green edges indicate functions that are promoted or increased in specific neutrophil subsets, while the red edges represent functions that are suppressed or decreased. On the right, neutrophil subsets related to different pathologies are shown, including cancer, infection, infarction, autoimmune diseases, and transplantation. This visual representation highlights the dynamic role of neutrophils and their functional shifts across physiological and pathological conditions, contributing to understanding their involvement in disease diagnosis, treatment, and prognosis. Abbreviations: NETs, Neutrophil Extracellular Traps; TANs, Tumor-Associated Neutrophils; PMN-MDSCs, Polymorphonuclear Myeloid-Derived Suppressor Cells; PR3, Proteinase 3; CD177, Cluster of Differentiation 177; TCR, T Cell Receptor; OLFM4, Olfactomedin 4.

Neutrophils exhibit remarkable diversity, which allows them to respond effectively to a variety of physiological and pathological conditions [14]. The heterogeneous population of neutrophils has been described by surface markers, density, maturity, function, and localization, according to different dimensions such as time, space, and body state [15,16,17,18,19]. Here, we summarize the phenotypic and functional heterogeneity of neutrophils in physiological and pathological conditions (Table 1 and Figure 1).

### 2.1. Neutrophil Heterogeneity in Homeostasis

Under physiological conditions, neutrophils demonstrate spatiotemporal heterogeneity through distinct developmental stages in bone marrow, circulation, and peripheral tissues.

In the bone marrow, the committed granulocytic progenitors derived from hematopoietic stem cells (HSCs) differentiate into myeloblasts, which in turn become promyelocytes, myelocytes, metamyelocytes, band cells, and segmented neutrophils [19]. Neutrophil surface markers change as neutrophils mature. In the steady-state state, specific surface markers of immature neutrophils included CD64, CD49d, and CXCR4, and mature neutrophils expressed CD10, CD16b, and CD35 [20,21]. Depending on the developmental maturity and activation status, neutrophils exhibit functional divergence. Immature neutrophils were deficient in the production of granules, which impair pathogen response [22]. Mature neutrophils exhibited stronger chemotactic migration with a complete granular system and receptor maturation [22].

In circulation, the phenotype of neutrophils undergoes distinct diurnal changes, which are known as neutrophil aging [23]. In mice, CD62L^low^ CXCR4^high^ aged neutrophil populations have been found in the circulation and returned to the bone marrow to be eliminated [24]. Aged neutrophils also expressed high levels of CD11b and CD49d, which related to adhesion to inflammatory endothelial cells [23]. Besides aged neutrophils, the subset of CD177^+^ neutrophils are constitutively present in the bloodstream [15]. This subset might play a protective role in inflammatory bowel disease by increasing bactericidal activity and IL-22 production [25]. In human peripheral blood, another study recently discovered an OLFM4^+^ neutrophil subpopulation [26]. OLFM4 was expressed in neutrophil-specific granules [26]. In mice, OLFM4 deletion enhanced innate immunity to resist bacteria [27,28,29]. In the circulation, Puellmann et al. demonstrated that 5–8% of human neutrophils expressed T cell receptors (TCR) [30]. The ligation of the TCR complex resulted in decreased neutrophil apoptosis and increased IL-8 secretion [30]. Sara Massena et al. described a proangiogenic neutrophil subpopulation (CD49d^+^ VEGFR1^high^ CXCR4^high^) that constituted 3–5% of circulating neutrophils in both humans and mice. This specialized subset localized to hypoxic tissues, engaged with vascular endothelium, and drove neovascularization through enhanced angiogenic signaling [31].

In addition to the bone marrow and circulation, neutrophils formed marginated pools and performed specific functions in the lung and spleen [15]. Puga et al. [32] identified a specific B-helper subset of neutrophils around the marginal zone (MZ) of the spleen, which induced Ig class switching, somatic hypermutation, and antibody production through activation of MZ B cells. B-helper neutrophil cells expressed more CD11b, CD24, CD27, CD40L, CD86, CD95, human leukocyte antigen-I (HLA-I), and HLA-II [32]. The marginated pool of the lung was thought to serve as a reservoir and transfer station of neutrophils via CXCR4 [33]. The lung provided a specialized niche for trapping circulating pathogens and their containment by preprepared marginal neutrophils [33]. Besides, the lung was the site of aged and activated neutrophils [33].

### 2.2. Neutrophil Heterogeneity in Disease

Based on physiological heterogeneity, neutrophils in different disease contexts differentiate into distinct subsets and have different functional characteristics [34,56].

Tumor-associated neutrophils (TANs) display remarkable heterogeneity within the tumor microenvironment (TME) [57]. Current classification proposes two polarization states, the antitumor N1 phenotype and protumor N2 phenotype [35]. Notably, these distinctions are currently limited to murine models and remain unverified in human systems [35]. TGF-β has been identified as a key driver of the N2 phenotype, while TGF-β blockade combined with IFN-β exposure promoted differentiation toward the N1 state [36,37]. TANs have dual functions in tumor biology; N1 has anti-tumor activity through direct or indirect cytotoxicity, while N2 has activities that promote tumor cell proliferation, promote angiogenesis, promote metastasis, and regulate immune responses [34].

Myeloid-derived suppressor cells are important contributors to tumor progression and metastasis [38]. Polymorphonuclear myeloid-derived suppressor cells (PMN-MDSCs), a pathologically activated neutrophil subset, demonstrate distinct immunomodulatory characteristics. These cells are phenotypically defined by the surface marker profile CD11b^+^ CD15^+^ CD14^-^ HLA-DR^-^ CD33^mild^ in humans, with upregulated expression of CD115 and CD244, accompanied by reduced CXCR1/CXCR2 chemokine receptors [39,40,41]. Functionally, PMN-MDSCs display enhanced immunosuppressive capacity: elevated arginase-1 and inducible nitric oxide synthase activity to impair T cell function; sustained ROS production that disrupts T cell receptor signaling; reduced phagocytic activity and chemotaxis [41,42]. Whether PMN-MDSCs represent a state in which neutrophils acquire immunosuppressive properties in tumors or develop as an independent lineage during myelopoiesis remains controversial.

Neutrophils have phenotypic and functional heterogeneity in infection. In sepsis, the expression of neutrophil CD11b, CD64, and ICAM-1 was upregulated, and the expression of CD62L, CXCR2, and CD16 was downregulated [43,44,45,46,47,48]. The functional changes of neutrophils in sepsis included emergency granulopoiesis, defective migration, delayed apoptosis, and enhancement of phagocytosis and oxidative burst during sepsis [45,49,50]. Neutrophils also demonstrated immunosuppressive potential through hydrogen peroxide-mediated inhibition of T cell proliferation [45,51,52].

In infarct disease, the functional polarization of neutrophils has been investigated. In myocardial infarction, Yonggang Ma et al. revealed dynamic neutrophil polarization regulated by damage-associated molecular patterns (DAMPs) [53]. Early-phase pro-inflammatory N1 neutrophils (Ly6G^+^ CD206^−^), activated through TLR-4 signaling, secreted proteolytic enzymes that contributed to ventricular wall thinning [53]. Conversely, late-phase anti-inflammatory N2 neutrophils (Ly6G^+^ CD206^+^) emerged with elevated expression of Arg1, IL-10, and Ym1, potentially facilitating tissue healing [53].

Neutrophil heterogeneity also manifests in autoimmune pathologies and transplantation. A unique PR3^+^ CD177^+^ subset has been implicated in granulomatosis with polyangiitis and polycythemia vera [54]. During islet transplantation, a circulating CD11b^+^ Gr-1^+^ CXCR4^hi^ neutrophil population was recruited by VEGF-A to deliver MMP-9, critically enhancing revascularization through extracellular matrix modulation [55].

## 3. Multi-Omics Research of Neutrophil Heterogeneity in Different Diseases

The advancement of omics technology has facilitated a deeper understanding of the fundamental characteristics of neutrophils, spanning from genes to transcription, proteins, metabolites, and even spatial localization in tissues.

### 3.1. Single Omics Unveil Neutrophil Heterogeneity

Neutrophil heterogeneity has been dissected through single-omics approaches, with each methodology unveiling unique molecular dimensions of functional diversity.

Genomic approaches have demonstrated the genetic basis of neutrophil heterogeneity. In autoimmune disorders, epigenome profiling revealed that lupus neutrophils and low-density granulocytes exhibited genome-wide demethylation, particularly at interferon signaling genes, suggesting epigenetically primed proinflammatory states through DNA methylome alterations [4]. In infection models, zebrafish neutrophils trained with Shigella developed H3K4me3 histone modifications at 1612 gene promoters, epigenetically enhancing microbial recognition and ROS production through chromatin remodeling [58]. Similarly, through chromatin immunoprecipitation sequencing (CHIP-seq), Jiang et al. demonstrated that neutrophils from juvenile idiopathic arthritis (JIA) patients exhibited enhancer-associated histone modifications [59].

Transcriptomics has emerged as a powerful tool to dissect neutrophil heterogeneity, especially single-cell transcriptomics. In cancer, TGF-β-mediated transcriptional reprogramming drove the transition from antitumor N1 to protumor N2 neutrophils, marked by distinct expression patterns in cytoskeletal organization, antigen presentation machinery, and chemokine networks [60]. Using single-cell transcriptomics, Gungabeesoon et al. identified immunotherapy-expanded Sell^hi^ state neutrophils with interferon signatures in murine models, demonstrating therapy-responsive transcriptional adaptation [61]. Dong et al. performed single-cell RNA sequencing (scRNA-seq) of cardiac CD45^+^ cells isolated from mouse myocardium [62]. They revealed an anti-inflammatory Ym-1^hi^ neutrophil subset and highlighted its critical role in myocardial protection in the early stages of myocardial ischemia-reperfusion injury [62].

Proteomic analyses have functionally validated neutrophil heterogeneity. Utilizing quantitative mass spectrometry, Lodge et al. demonstrated hypoxia drove a destructive hypersecretory neutrophil phenotype with enhanced capacity for endothelial injury [63]. The corresponding signature of neutrophil degranulation and vascular injury was identified in the plasma of patients with chronic obstructive pulmonary disease [63].

Metabolomics decodes the functional change of neutrophils. Using metabolomics, Li et al. identified widespread dysregulation of neutrophil metabolism with COVID-19 progression, including in amino acid, redox, and central carbon metabolism [64]. They demonstrated that neutrophils displayed a reduction in GAPDH activity in severe COVID-19 and that GAPDH inhibition promoted neutrophil extracellular trap formation [64].

### 3.2. Unraveling Neutrophil Diversity: Insights from Multi-Omics Study

Single-omics data sometimes cannot explain complex biological phenomena in depth. Through integrating multiple omics data, these data from different molecular levels can be mutually verified. It is beneficial to complement each other and is more conducive to a more comprehensive understanding of biological systems. The comprehensive integration of multi-omics approaches has significantly improved our understanding of neutrophil heterogeneity in different diseases, revealing intricate functional reprogramming in a pathological context.

Through integrated transcriptomic-proteomic analysis during neutrophil differentiation, Hoogendijk et al. reported the dynamic changes of the five stages of neutrophil development and differentiation in bone marrow and blood [65]. They demonstrated that a general decrease in mitochondrial, metabolic, and protein synthesis processes occurs during neutrophil maturation [65]. But at the same time, neutrophils acquired a strong motile capacity and produced cytotoxic proteins, which were stored in intracellular vesicles [65]. In particular, this study redefined the identity of neutrophil precursor cells by classifying their maturation stages based on their proliferative and molecular properties [65]. Evrard et al. combined mass cytometry (CyTOF) with transcriptional analysis to resolve neutrophil precursor heterogeneity in bone marrow [66]. Using CyTOF, they revealed differentially expressed markers between proliferative and non-proliferative neutrophils in the Fucci-(S-G2-M) mouse. Non-proliferating neutrophils highly expressed Ly6G and CXCR2, whereas proliferating neutrophils were Ly6Glo CXCR2− and were positive for cKit and CXCR4 [66]. A potential proliferative granulocyte precursor was identified, termed preNeus [66]. Transcriptomic profiling and functional analysis revealed that the proliferative program of preNeus was substituted by a gain of migratory and effector function as they mature [66]. Furthermore, preNeus expanded under microbial and tumoral stress [66]. In summary, they identified specific subsets of neutrophil precursors that proliferated under homeostasis and stress [66]. The integration of metabolomics with transcriptomics by Hsu et al. uncovered functional heterogeneity within circulating neutrophil populations [67]. Their multi-omics characterization identified immature low-density neutrophils (iLDNs) as a metabolically plastic subset capable of maintaining NETosis through glutamate/proline catabolism when glucose-deprived [67]. NETosis is an important neutrophil function that promotes breast cancer liver metastasis [67]. This metabolic flexibility enabled iLDNs to exert protumor metastatic functions in the absence of glucose, illustrating how microenvironment-driven metabolic adaptation contributes to neutrophil functional diversification [67]. Using multi-omics approaches, Wang et al. identified BHLHE40-driven pro-tumor neutrophils with hyperactivated glycolysis in the pancreatic tumor microenvironment [68]. They identified four major subpopulations in pancreatic tumor microenvironment through scRNA seq: a terminally differentiated pro-tumor subpopulation (TAN-1) associated with poor prognosis, an inflammatory subpopulation (TAN-2), a population of transitional stage that has just migrated to tumor microenvironment (TAN-3), and a subpopulation preferentially expressing interferon-stimulated genes (TAN-4) [68]. Trajectory analysis positioned TAN-1 as the terminal differentiation state with progressive glycolytic activation along the neutrophil maturation path [68]. The multi-omics strategy through quantitative proteomic validation confirmed glycolytic pathway enrichment in TANs [68]. Metabolomic profiling of neutrophil lysates further substantiated this metabolic reprogramming through elevated glycolytic intermediates [68]. Through single-cell regulatory network inference and clustering (SCENIC) analysis, they identified BHLHE40 as a key transcriptional driver of TAN-1 differentiation, while functional validation of LDHA established its critical role in immunosuppression and tumor promotion [68]. Myocardial ischemia-reperfusion injury (MIRI) is a major obstacle to the success of cardiac reperfusion therapy, whereas increased neutrophil infiltration is a hallmark of MIRI. Mi Zhou et al. [69] employed a triad of single-cell transcriptomics, metabolomics, and proteomics to decode neutrophil heterogeneity in traumatic brain injury (TBI). Their integrated approach identified FOXO1^high^ neutrophils as dual-effect cells driving acute neuroinflammation while predisposing to post-TBI depression [69]. Multi-omics correlation revealed that FOXO1 activation coordinated a metabolic shift from glycolysis to aerobic oxidation, evidenced by 58 altered metabolites and enhanced ATP production [69].

### 3.3. Spatial Omics Reveal Location Characteristics of Neutrophils

Spatial omics offer transformative perspectives on neutrophil heterogeneity by mapping their molecular and functional diversity within tissue microenvironments. Recent advances in spatial omics technologies have provided unprecedented insights into the heterogeneity of neutrophils in the tumor microenvironment.

Wang et al. employed spatial transcriptomics to map pancreatic ductal adenocarcinoma (PDAC) tissues, identifying tumor-specific glycolytic activation at neutrophil-enriched sites compared to stromal regions, suggesting a metabolic shift toward glycolysis in neutrophils under PDAC microenvironmental pressures [68]. Expanding the spatial understanding of neutrophil dynamics, Rao et al. integrated spatial transcriptomics and multiplex immunohistochemistry in gallbladder cancer liver invasion (GBC-LI), revealing that neutrophils constituted 25% of the immune infiltrate in adjacent liver tissues and exhibited preferential spatial accumulation at invasive margins, implicating their role in fostering metastatic progression [70]. Further spatial dissection by Yongjie Xie et al. has demonstrated a strong correlation between EHF gene expression and the spatial distribution of CXCR2^+^ neutrophils through transcriptomics analysis [71]. The expression of EHF in pancreatic cancer tissues was closely related to chemotaxis, infiltration, and accumulation of CXCR2^+^ neutrophils, showing a significant negative correlation between them [71]. Furthermore, the spatial analysis revealed that CXCR2^+^ neutrophils are located distally from high-EHF tumor cells but proximally to low-EHF tumor cells [71]. Melissa S F Ng et al. employed an integrative approach combining scRNA-seq, spatial transcriptomics, and flow cytometry to investigate the heterogeneity of TANs in murine models [72]. Spatial resolution uncovered three distinct tumor-adapted neutrophil clusters (T1-T3) with microenvironment-specific localization patterns [72]. Pro-tumorigenic T3 neutrophils preferentially localized to hypoxic tumor cores, while stromal-margin regions harbored neutrophils associated with epithelial-mesenchymal transition pathways [72].

Contextualizing the microanatomical localization of cells helps to understand the behavior of cells. The spatial omics approaches collectively emphasize the critical role of tissue architecture in shaping neutrophil functional states and distribution patterns, establishing neutrophil plasticity as a spatially encoded phenomenon.

## 4. Machine Learning in Multi-Omics Analysis Unveils Neutrophil Heterogeneity

**Figure 2:**
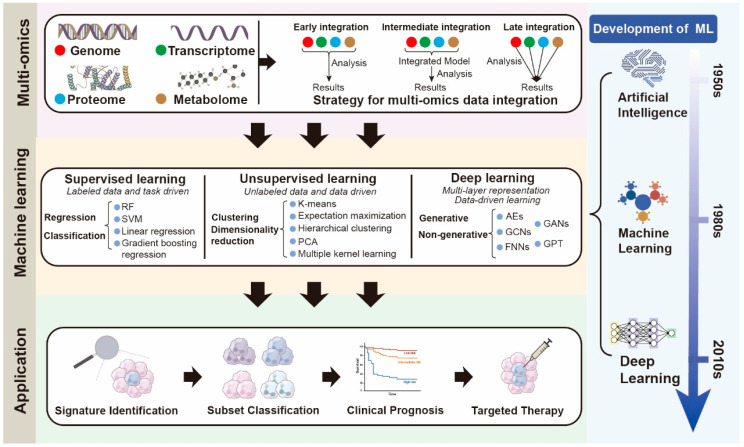
**Comprehensive Pipeline for Multi-Omics Integration and Machine Learning-Based Neutrophil Classification:** this schematic illustrates an integrative workflow combining multi-omics data acquisition, integration strategies, and the evolution of machine learning approaches to generate clinically relevant neutrophil classifications and practical applications. In the top panel, four major omics layers—genomics, transcriptomics, proteomics, and metabolomics—are shown, which can be integrated using early, intermediate, or late integration strategies. The middle panel summarizes the spectrum of machine learning paradigms: supervised learning (regression and classification), unsupervised learning (clustering and dimensionality reduction), and deep learning (generative and non-generative frameworks), contextualized within the historical development of artificial intelligence, machine learning, and deep learning. The bottom panel demonstrates four major translational applications derived from this pipeline: (1) signature identification of key molecular features, (2) subset classification of neutrophil populations, (3) clinical prognosis prediction, and (4) targeted therapy discovery. Together, this pipeline underscores the synergistic role of multi-omics integration and machine learning in elucidating neutrophil heterogeneity and advancing precision medicine. Abbreviations: ML, Machine Learning; AI, Artificial Intelligence; DL, Deep Learning; RF, Random Forest; SVM, Support Vector Machine; PCA, Principal Component Analysis; AE, Autoencoder; GAN, Generative Adversarial Network; GCN, Graph Convolutional Network; FNN, Feedforward Neural Network; GPT, Generative Pretrained Transformer.

The high-dimensional data in multi-omics studies require complex computational analysis. Thus, the field of artificial intelligence (AI) and its subfields, especially ML, have become important tools to assist multi-omics analysis [73]. Heterogeneous omics datasets spanning genomics, proteomics, and metabolomics can be synergistically analyzed through machine learning computational frameworks to discover potential data characteristics. The convergence of multi-omics data integration with ML represents a paradigm shift in systems biology research and precision medicine implementation, facilitating a deeper understanding of neutrophil heterogeneity and its implications for health and disease. (Figure 2)

### 4.1. Scope and Development of ML

The field of AI originated in the mid-1950s as an interdisciplinary domain focused on developing computational systems capable of performing tasks requiring human-like cognitive functions [74]. ML emerged as a distinct AI subfield during the 1980s, enabling systems to improve task performance through data pattern recognition rather than explicit programming [75]. ML encompasses multiple methodological frameworks, including supervised, unsupervised, semi-supervised, and reinforcement learning approaches, each addressing different categories of computational challenges [76]. Recent technological advancements have witnessed the rise of deep learning (DL) as a specialized ML approach utilizing multi-layered neural architectures [77]. These approaches demonstrate enhanced capability in autonomously extracting discriminative features from complex datasets, significantly expanding the range of addressable problems in AI applications [78]. Notably, the integration of AI with multi-omics data analysis has opened new avenues for exploring biological complexities, particularly in understanding neutrophil heterogeneity. By leveraging machine learning techniques on genomics, proteomics, and metabolomics data, researchers are uncovering distinct subpopulations of neutrophils and their functional implications, thereby providing insights into immune responses and disease pathogenesis.

### 4.2. Types of Multi-Omics Datasets and Strategy for Integration

Advances in high-throughput cell biology techniques allow researchers to study the distribution of various types of biomolecules. Large-scale studies of genomes, transcriptomes, proteomes, metabolomes, lipidomes, etc., have generated a large amount of data related to these “groups”, also known as “multi-omics” datasets.

The integration of heterogeneous multi-omics datasets forms the foundation of ML models. ML approaches in multi-omics analysis rely on the characteristics of the datasets utilized. Different types of multi-omics datasets and their features have different biological significance in capturing the heterogeneity of biological samples. Genomic data reveal genetic variation among individuals and within heterogeneous tissues such as tumors [79]. In the realm of medical research, genomics focuses on identifying genetic variants associated with disease, response to treatment, or future patient prognosis [80]. Genome sizes can range from 4000 bases to 670 Gb [81]. The human genetic genome has two copies of 3.2 Gb each [81]. Epigenomics focuses on genome-wide characterization of reversible modifications of DNA or DNA-associated proteins, such as DNA methylation or histone acetylation [82]. Covalent modifications of DNA and histones are major regulators of gene transcription and subsequently of cellular fate [83]. Those modifications can be influenced by both genetic and environmental factors, can be long-lasting, and are sometimes heritable [84]. Transcriptome data offer a direct means to investigate gene and transcript expression, enabling both qualitative and quantitative analyses. Qualitatively, it allows the identification of expressed transcripts, novel splice junctions, and RNA editing events [79]. Quantitatively, it provides insights into the abundance of each transcript, thereby facilitating a comprehensive understanding of gene expression dynamics [79]. Proteomic data identify and quantify proteins in samples, as well as determine protein structure, function, and interactions [85]. Metabolomics is a comprehensive analysis of metabolites in a specimen to measure the internal biochemical activity of cellular processes [86].

Multi-omics data integration can be approached through various strategies, each offering distinct advantages based on the nature of the data and the integration goals [87,88]. In early integration, features from different omics modalities (e.g., genomics, transcriptomics, proteomics) are concatenated into a single feature vector before being input into the model. This approach assumes that the modalities are comparable, allowing the model to learn the relationships between them directly. However, more advanced methods utilize intermediate integration, where modalities are treated as separate entities initially. These approaches learn inter-modality relationships by generating a shared latent space or an integrated model, which facilitates a more nuanced understanding of the interactions between different biological layers. Late integration, on the other hand, involves training separate models for each modality independently, with the final results being aggregated to produce a comprehensive outcome. This method is particularly useful when the modalities vary significantly in their characteristics or data distributions.

### 4.3. ML Methods and Application in Multi-Omics Analysis

In multi-omics studies, ML methods can be categorized into supervised and unsupervised learning based on data labeling availability [89].

Supervised learning establishes input-output mappings through labeled training data, where algorithms discern relationships between input features and target variables to predict outcomes for new inputs [74]. In multi-omics analysis including classification and regression tasks, through a variety of algorithm implementations, including support vector machines (SVM), neural networks and decision trees, and random forest (RF) [90]. Particularly, RF is a commonly used algorithm in multi-omics studies of neutrophil heterogeneity. This algorithm captures complex data patterns by majority voting in multiple decision trees while providing feature importance measures for biological prioritization that can identify key biomarkers [91]. Conversely, unsupervised learning extracts latent patterns from unlabeled data through self-organizing algorithms [74]. The main tasks in unsupervised learning include clustering and dimensionality reduction, which aim to uncover hidden structures within the data [92]. Clustering divides data into groups of similar objects based on their intrinsic properties without prior knowledge of group labels. Classical algorithms include K-means, hierarchical clustering, and expectation maximization [93]. Dimensionality reduction addresses high-dimensional data challenges through algorithms like principal component analysis (PCA) that project data onto orthogonal eigenvectors [94], factor analysis that identifies latent variables capturing common variance [95], and multiple kernel learning that enables non-linear feature space transformations [96]. Deep learning is one of the recent advances in machine learning. The ability of DL to capture nonlinear features, interaction effects, and hierarchical representations through multilayer neural network architectures without kernel tricks is one of the main advantages of DL generally observed in biological systems [97]. Deep learning-based approaches for multi-omics data integration can be broadly classified into non-generative and generative methods. Non-generative methods include feedforward neural networks (FNNs), graph convolutional networks (GCNs), and autoencoders (AEs), while generative methods encompass variational methods, generative adversarial networks (GANs), and generative pretrained transformers (GPT) [87].

The development of high-throughput omics technology has greatly improved our ability to study biological systems at the molecular level [98]. However, each omics technique provides only a limited perspective on the underlying biological processes. Integrating different omics data will facilitate a more comprehensive and detailed understanding of diseases and phenotypes. Multi-omics integrated analysis models based on different statistical methods and machine learning principles have been developed for disease classification and prognostic diagnosis, especially in cancer.

Nguyen et al. developed PINSPlus, an unsupervised machine learning method for tumor subtype discovery that does not rely on prior clinical knowledge [99]. By assessing the stability of subtypes under small perturbations in data, PINSPlus effectively identifies both known and novel cancer subtypes with significant survival differences, outperforming existing methods [99]. Cancer Integration via Multikernel Learning (CIMLR) is a novel method developed for multi-omic cancer subtyping that integrates diverse data types—gene expression, methylation, point mutations, and copy number changes—to reveal biologically meaningful molecular subtypes [100]. CIMLR utilizes a multi-kernel approach, incorporating multiple Gaussian kernels to assess similarity across data types, and ensures robustness by using a block-structured similarity matrix for dimension reduction and clustering [100]. Applied to multi-omics data across 36 cancer types, CIMLR demonstrates significant improvements in both computational efficiency and the ability to uncover subtypes that show meaningful differences in patient survival [100]. iCluF is an unsupervised multi-omics integration method that combines mRNA, miRNA, and DNA methylation data through iterative matrix fusion and message passing [101]. It constructs pairwise patient similarity matrices for each omics type and refines them via iterative updates to generate a final integrated matrix, which is then used to cluster patients into subtypes [101].

With the adoption of deep learning as a new method in medical applications [102], several studies have explored the use of deep learning in the analysis of multi-omics data integration.

Subtype-GAN employs a multi-input multi-output neural network combined with adversarial learning to integrate diverse omics data [103]. By extracting shared latent representations, it uses consensus clustering and Gaussian Mixture Models to identify clinically meaningful cancer subtypes from complex multi-omics profiles [103]. Benkirane et al. introduced a new customizable architecture for multi-omics integration, called CustOmics [104]. CustOmics exemplifies how machine learning advances multi-omics integration by leveraging representation learning to map heterogeneous omics data into a shared latent space [104]. CustOmics employs a two-phase deep learning strategy: first training modality-specific networks independently, then integrating them jointly to model cross-omics interactions [104]. This hybrid approach enhances flexibility, interpretability via Shapley values, and predictive performance, highlighting deep learning’s capacity to capture complex biological relationships across diverse omics layers [104]. Cross-omics Linked unified embedding with Contrastive Learning and Self Attention (CLCLSA) is a deep learning method designed for integrating incomplete multi-omics data [105]. It consists of three key components: cross-omics autoencoders, contrastive learning, and self-attention mechanisms [105]. Using complete multi-omics data as supervision, the model learns cross-omics feature representations and reconstructs missing modalities through modality-specific embeddings [105]. Contrastive learning maximizes mutual information between different omics layers, while feature-level and omics-level self-attention dynamically identify the most informative features [105].

In conclusion, integrated multi-omics approaches, especially those using machine learning and deep learning, have shown superior performance in disease classification and prognosis. These approaches enhance our understanding of biological complexity and heterogeneity and provide a robust framework for advancing precision medicine and improving clinical decision-making.

### 4.4. Application of ML in Multi-Omics Analysis of Neutrophil Heterogeneity

ML has significantly advanced the study of neutrophil heterogeneity by participating in different steps of multi-omics analysis (Table 2).

Using RF analysis, Meyer et al. investigated the association between CD79b expression in peripheral blood neutrophils and melanoma incidence [106]. Utilizing CyTOF, they profiled surface marker expression in blood neutrophils from melanoma patients and identified a CD79b^+^ neutrophil subset [106]. Variable importance analysis revealed CD79b as a critical marker for distinguishing melanoma patients, supported by flow cytometry validation [106]. These findings highlight CD79b^+^ neutrophils as potential biomarkers for early melanoma detection. In a multi-omics investigation of neutrophil heterogeneity in gastric cancer (GC), Tang et al. identified neutrophil-associated gene signatures and developed a robust prognostic model through ML algorithms [107]. Utilizing non-negative matrix factorization (NMF) clustering, three distinct molecular subtypes were distinguished [107]. Correlating with reduced survival, cluster 3, characterized by increased neutrophil infiltration, exhibited enrichment in pathways related to neutrophil chemotaxis, extracellular matrix remodeling, and recruitment [107]. Using RF, they developed a robust prognostic model validated in multiple independent cohorts, in which high-risk patients consistently demonstrated inferior survival outcomes [107]. This study systematically reveals the heterogeneity of neutrophils in GC and provides a stratification tool for GC prognosis. In another multi-omics investigation of long COVID patients following Omicron infection, Lin et al. demonstrated distinct neutrophil functional polarizations through integrated transcriptomic and proteomic profiling combined with ML [108]. Using multi-omics stratification, their analysis of 66 participants, including 22 long COVID (LC) cases, uncovered two neutrophil-driven LC subtypes: NU-LC with upregulated neutrophil degranulation and activation and ND-LC with downregulated neutrophil function [108]. Utilizing five blood-derived gene markers (ABCA13, CEACAM6, CRISP3, CTSG, and BPI), the RF model achieved exceptional classification performance in distinguishing NU-LC from other LC patients [108]. Jin et al. demonstrated a comprehensive research workflow integrating ML, multi-omics analysis, and experimental validation to investigate the relationship between NETs-related genes and colorectal cancer (CRC) [109]. They identified three key NET-related genes (TIMP1, F3, and CRISPLD2) by taking the intersection of three ML screening results (SVM-RFE, RF, and COX regression) [109]. Based on these three genes, patients were robustly stratified into two distinct prognostic subgroups by unsupervised clustering (k = 2) and principal component analysis [109]. Single-cell transcriptomics showed an elevated signature of NETs in tumor tissues, while TIMP1 showed a strong association with neutrophils in both scRNA-seq and bulk RNA-seq data [109]. The results showed that TIMP1 is associated with VEGF-A expression and enrichment of the ferroptosis pathway in neutrophils [90]. In addition, biological experiments confirmed that TIMP1 promoted the proliferation, invasion, and metastasis of colorectal cancer [109]. This study highlights how ML, combined with multi-omics, identifies pathogenic genes in neutrophils and uncovers the role of the highly TIMP1-expressing neutrophil subset in CRC. Recent advances in multi-omics profiling have provided valuable insights into neutrophil heterogeneity regarding age- and sex-related biology. Integrating transcriptomic, metabolomic, and lipidomic analyses of primary mouse neutrophils, Lu et al. identified distinct regulatory patterns associated with organismal aging and biological sex, including chromatin remodeling dynamics and differences in NETosis activation [110]. Seven ML algorithms demonstrated the ability to identify features associated with age-dependent and sex-dimorphic gene expression patterns, achieving classification accuracies of over 64% compared to random chance (50%) [110]. AI-based feature importance analysis further highlighted correlations between CpG content, transcription factor target specificity, and sex-biased neutrophil functions, though such computational predictions require experimental validation to establish causality [110]. The analysis of the glioblastoma-associated neutrophil (GBMAN) subpopulation by Yang et al. may provide new insights and opportunities for glioblastoma (GBM) immunotherapy [111]. By integrating large-scale scRNA-seq data, neutrophils were divided into four distinct subtypes [111]. Among them, the VEGFA^+^ GBMAN subset showed reduced inflammatory response characteristics and tended to interact with stromal cells [111]. They also used ten machine learning algorithms to develop a robust predictive risk model called “VEGFA^+^ neutrophil-related signatures” (VNRS), and the VNRS model genes were validated at the RNA and protein levels [111]. The VNRS model had higher accuracy than previously published risk models and was an independent prognostic factor [111]. There were significant differences between high-risk and low-risk VNRS score groups in terms of immunotherapy response, tumor microenvironment interaction, and chemotherapy efficacy [111]. To explore the role of TANs in tumor immunity and their response to immune checkpoint inhibitors (ICIs), Zhang et al. identified a CD44_NEU subset of neutrophils expressing high levels of CD44 in primary GC associated with treatment response to ICIs, which are abundant during tumor progression and have a significant impact on the GC immune microenvironment [112]. Lasso, Univariate, RF, and Boruta ML algorithms from the GeneSelectR package were used to identify the core genes of CD44_NEU. ML analysis revealed 22 core genes associated with CD44_NEU that affected inflammation, proliferation, migration, and oxidative stress [112]. This study highlights the heterogeneity of TANs, especially the influence of CD44_NEU on immunotherapy outcomes, paving the way for personalized treatment strategies [112].

The integration of ML with multi-omics frameworks has advanced neutrophil heterogeneity research by enabling high-dimensional decoding of dynamic subpopulations across diseases. AI-driven models reveal non-linear relationships and latent molecular networks missed by conventional methods, while correlating transcriptomic, proteomic, and epigenetic variations with clinical phenotypes. This synergy enhances biomarker discovery, therapeutic target identification, and AI-powered patient stratification for precision medicine.

### 4.5. Challenges in Multi-Omics Analysis Using ML

Multi-omics analysis leverages machine learning to uncover biological insights [113]. However, this approach faces significant challenges across data, methodology, ethics, and practice.

#### 4.5.1. Data-Related Challenges

The integration of multi-omics data derived from diverse high-throughput platforms presents inherent challenges due to their heterogeneous nature [114]. Picard et al. pointed out that data heterogeneity increases the complexity of the integration strategies [115]. For instance, transcriptomic data typically undergoes RNA-seq normalization while proteomic data employs mass spectrometry-specific scaling methods, resulting in distinct ranges and distribution patterns that require alignment prior to integration.

Data quality is also a major issue. Noise, missing values, and batch effects can significantly affect the analysis results [116]. The challenge arises from modality-specific data characteristics, where certain omics layers like metabolomics frequently generate sparse datasets due to technical limitations (e.g., values below detection thresholds being recorded as null) [117]. This necessitates tailored preprocessing strategies, including omics-specific imputation methods and outlier detection algorithms, to ensure data quality before cross-modal integration [118,119].

#### 4.5.2. Methodological Challenges

High-dimensional data are the primary problem, and the number of features far exceeds the number of samples, which easily leads to model overfitting [120]. Most multi-omics datasets suffer from the classical “curse of dimensionality” problem, i.e., having much fewer observation samples than multi-omics features [121]. The resulting high-dimensional space often contains correlated features that are redundant and can mislead the algorithm training. Therefore, techniques such as dimension reduction or feature selection need to be adopted to deal with it [122,123,124]. Meng et al. offer a review of these methods from the perspective of multi-omics data analysis [125].

Researchers need to select the appropriate ML algorithm based on different data types and the applicability of research questions. Nicora et al. described the tools presenting the most promising innovations regarding the integration of heterogeneous data and the ML methodologies that tackled the complexity of multi-omics data [73].

The analysis of multi-omics datasets presents significant challenges in identifying biologically meaningful patterns and distinguishing true associations from noise [126,127,128,129,130]. In the field of biomedicine, model interpretability is paramount [131]. Traditional “black-box” AI models frequently fail to provide the mechanistic insights necessary for biological interpretation, thereby complicating the validation of findings.

#### 4.5.3. Ethical and Practical Challenges

The ethical and practical challenges of multi-omics analysis further complicate the research landscape.

At the data collection and analysis level, core ethical concerns include algorithmic bias, often stemming from unrepresentative training data sets or flawed algorithm design, which can lead to discriminatory results [132]. In clinical applications, it is important to obtain informed consent from study participants to ensure that they understand how the data will be used and the potential risks. In addition, data sharing has the potential to compromise patient privacy, necessitating anonymization schemes. Transparency and interpretability of AI and ML models are critical to promoting trust and accountability, as algorithms do not fully mimic real biological processes, which can erode clinician confidence and hinder clinical adoption [116]. For now, AI should be used as a supportive tool for clinicians to critically evaluate algorithm output by considering contextual relevance, underlying assumptions, and potential biases to enable more effective yet human-centered decision making [117]. At the societal level, ethical implications extend to systemic issues such as equitable access to care and the distribution of medical liability among stakeholders [114].

In practice, the implementation of ML for multi-omics analysis incurs significant computational and data storage costs [133]. The high expense associated with advanced omics technologies limits accessibility for many researchers [131]. Furthermore, the computational complexity of large-scale datasets imposes heightened demands on high-performance computing resources [131]. Addressing these practical challenges is crucial for fostering a more inclusive and representative research ecosystem.

## 5. Conclusions

Neutrophils are now recognized as a dynamic immune subpopulation with phenotypic and functional diversity under physiological and disease conditions [10]. However, it is not clear how to understand the relationship between neutrophil subsets. How do different neutrophil subsets evolve in homeostasis? How do diseases affect the transformation of specific neutrophil populations? How can neutrophil subsets be distinguished more precisely? Features at the level of genes, transcription, proteins, and metabolic molecules may represent core properties of neutrophils.

The integration of ML and multi-omics techniques has revolutionized our understanding of neutrophil heterogeneity, revealing previously unrecognized functional subsets and the molecular mechanisms underlying their plasticity. Single-cell transcriptomics have resolved the dynamic transcriptional state during neutrophil maturation and activation [66], while spatial omics have mapped microenvironment-specific functional adaptations [71]. ML algorithms further decode high-dimensional datasets to identify biomarkers and prognostic signatures. These advances highlight the transformative potential of ML in combination with multi-omics analysis to connect molecular insights with clinical outcomes [106]. However, significant challenges remain. First, substantial data heterogeneity across omics layers complicates integration efforts [115]. Transcriptomic and proteomic datasets frequently demonstrate discordant biological dynamics due to post-transcriptional regulatory mechanisms, requiring sophisticated standardization frameworks to reconcile platform-specific normalization methods [116]. Second, the biological interpretation of machine learning-derived patterns remains constrained by algorithmic opacity. While contemporary feature importance metrics can identify putative biomarkers such as differentially expressed genes or metabolites, these computational predictions require systematic experimental validation to establish causal relationships and elucidate underlying biological mechanisms [131]. Finally, significant ethical and practical barriers persist in translating omics technologies to resource-limited environments. With the development of multi-omics and AI, their integration will not only improve our understanding of neutrophil biology and redefine the role of neutrophils in pathophysiology, but also bridge molecular discovery and precision medicine.

Looking ahead, there are several promising directions that could advance our understanding of neutrophil biology and improve the clinical translation of multi-omics research. First, more sophisticated data integration methods are needed to address the challenge of data heterogeneity. Multi-omics platforms need to be harmonized, and new algorithms should be developed to account for the complexity and noise inherent in these datasets. Second, the interpretability of ML models must be enhanced. Third, given the variability in neutrophil subsets across different diseases, future research should focus on the longitudinal tracking of neutrophil populations over time, particularly in response to treatments. This would enable a better understanding of how these cells contribute to disease progression and recovery.

Finally, the clinical application of multi-omics technologies will require closer collaboration between researchers, clinicians, and bioinformaticians to ensure that molecular insights are translated into actionable clinical strategies. Leveraging ML alongside multi-omics in precision medicine could lead to the identification of novel therapeutic targets and the development of more effective treatment plans tailored to individual patients.

## Figures and Tables

**Table 1:** Neutrophil Functional and Phenotypic Heterogeneity in Homeostasis and Disease.

Context	Subpopulation	Phenotype	Function Change	References
Homeostasis				
Bone Marrow	Immature Neutrophils	CD64^+^, CD49d^+^, CXCR4^+^	Reduced granule production.	[20,21,22]
	Mature Neutrophils	CD10^+^, CD16b^+^, CD35^+^	Strong chemotaxis, complete granule system, receptor maturation.	[20,21,22]
Circulation	Aged Neutrophils	CD62L^low^, CXCR4^high^, CD11b^+^, CD49d^+^	Enhanced adhesion, return to bone marrow for clearance.	[23,24]
	CD177^+^ Neutrophils	CD177^+^	Increased bactericidal activity, IL-22 production.	[15,25]
	OLFM4^+^ Neutrophils	OLFM4^+^	Modulates innate immunity, OLFM4 deletion enhanced antibacterial defense.	[26,27,28,29]
	TCR^+^ Neutrophils	TCR^+^	Reduced apoptosis, increased IL-8 secretion.	[30]
	Proangiogenic Neutrophils	CD49d^+^, VEGFR1^high^, CXCR4^high^	Hypoxic tissue localization, neovascularization.	[31]
Organs	Spleen B-helper Neutrophils	CD11b^+^, CD24^+^, CD27^+^, CD40L^+^, CD86^+^, HLA-I/II^+^	Induce antibody production via MZ B cells.	[32]
	Lung Marginated Neutrophils	CXCR4^+^	Pathogen containment, reservoir.	[33]
**Disease**				
Cancer	N1 TANs	TGF-β blockade ^+^ IFN-β exposure	Antitumor: Direct cytotoxicity, immune activation	[34,35,36,37]
	N2 TANs	TGF-β-driven	Protumor: Angiogenesis, metastasis, immunosuppression	[34,35,36,37]
	PMN-MDSCs	CD11b^+^ CD15^+^ CD14^−^ HLA-DR^−^ CD33^mild^	Immunosuppression: Arg-1, iNOS, ROS ↑, T cell function ↓, reduced phagocytic activity and chemotaxis.	[38,39,40,41,42]
Infection	Neutrophils in infection	CD11b ↑, CD64 ↑, ICAM-1 ↑, CD62L ↓, CXCR2↓, CD16 ↓	Defective migration, delayed apoptosis, enhanced phagocytosis/oxidative burst, T cell suppression	[43,44,45,46,47,48,49,50,51,52]
Cardiovascular disease	N1 Neutrophils	Ly6G^+^, CD206^−^	TLR-4 activation, protease secretion.	[53]
	N2 Neutrophils	Ly6G^+^, CD206^+^	Arg1, IL-10, Ym1 ↑	[53]
Autoimmunity	PR3^+^ CD177^+^ Neutrophils	PR3^+^, CD177^+^	Pathogenic role in vasculitis and polycythemia.	[54]
Transplantation	Pro-revascularization	CD11b^+^, Gr-1^+^, CXCR4^high^	VEGF-A recruitment, MMP-9 delivery.	[55]

↑ = increased, upregulated or promotion; ↓ = decreased, downregulated or inhibition. Abbreviations: CD, cluster of differentiation; CXCR, CXC chemokine receptors; OLFM4, olfactomedin4; IL, interleukin; TCR, T cell receptor; VEGFR1, vascular endothelial growth factor receptor 1; HLA, human leukocyte antigen; MZ, marginal zone; IFN-β, interferon-β; TGF-β, transforming growth factor-β; PMN-MDSC, polymorphonuclear myeloid-derived suppressor cells; Arg1, arginase 1; iNOS, inducible nitric oxide synthase; ROS, reactive oxygen species; ICAM-1, intercellular cell adhesion molecule-1. PR3, Proteinase 3; VEGF-A, vascular endothelial growth factor-A; MMP-9, matrix metallopeptidase 9.

**Table 2:** Comparison of Machine Learning Models and Performance in Neutrophil Heterogeneity Studies.

First Author (Year)	Context	ML Methods	Omics Types	Dataset	Performance	Application and Key Finding
Meyer M.A. et al. (2023) [106]	Melanoma	RF	Bulk RNA-sequencing, CyTOF	GSE154777, GSE139324	Biomarker ranking	Variable importance analysis prioritized CD79b as a diagnostic biomarker. CD79b^+^ neutrophils as a potential biomarker for early melanoma detection.
Tang G. et al. (2024) [107]	Gastric Cancer	RF	Bulk RNA sequencing scRNA-seq	GSE15459, GSE57303, GSE62254, GSE84437, GSE26253, and a combined cohort of all GEO data.	Identified the most important genes contributing to the prognostic score; C index= 0.827	Establishment of a stratified prognostic model for GC patients.
Lin K. et al. (2024) [108]	Long COVID	RF	Bulk RNA sequencing CyTOF	Long COVID cohort	Average ROC AUC = 0.95	A model for identifying NU-LC in long COVID patients was developed.
Jin Y. et al. (2025) [109]	Colorectal Cancer	SVM-RFE, RF, Univariate.	Bulk RNA sequencing scRNA-seq	CRC cohort	Gene importance ranking	TIMP1 was identified as a key NET-related gene. Revealing TIMP1^+^ neutrophil subset in CRC progression.
Lu R.J. et al. (2021) [110]	Age and Sex	NNET, RF, gradient boosting, SVM, LDA, cTree, LogReg.	Bulk RNA sequencing Metabolomics Lipidomics ATAC-seq proteomics	BioProject PRJNA630663, GSE1248296.	Age-related: Accuracy >64%, Sex-related: Accuracy >70%	Predicting characteristics of gene expression patterns in neutrophils regarding age and sex.
Yang Y. et al. (2025) [111]	Glioblastoma	stepwise Cox, CoxBoost, Lasso, Ridge, Elastic Net, survival-SVMs, SuperPC, Generalized Boosted Regression Models, plsRcox, RSF.	scRNA-seq	EGAS00001004656, EGAS00001005300, 9 GSE datasets, and TCGA-GBM.	C-index = 0.643 (optimal model); AUC (1-year, 2-year, 3-year) in TCGA-GBM: 0.766, 0.763, 0.803	Developing VEGFA^+^ neutrophil derived VNRS model for predicting risk in GBM.
Zhang J. et al. (2024) [112]	Gastric Cancer	LASSO, Univariate, RF, Boruta.	scRNA-seq	GSE163558, GSE183904, GSE205506, GSE207422, and TCGA-STAD.	22 key genes selection	Identified CD44_NEU subset with 22 core genes regulating inflammation, proliferation, migration, and oxidative stress, predictive of ICI response.

Abbreviations: ML, Machine Learning; RF, Random Forest; CyTOF, Mass Cytometry; GC, gastric cancer; SVM-RFE, Support Vector Machine-Recursive Feature Elimination; scRNA-seq, single-cell RNA sequencing; NET, Neutrophil Extracellular Traps; CRC, Colorectal Cancer; NNET, neural networks; SVM, Support Vector Machines; LDA, Linear Discriminant Analysis; cTree, conditional inference Trees; LogReg, Regularized Logistic Regression; plsRcox, partial least squares Cox; RSF, Random Survival Forest; GBM, Glioblastoma; VNRS, VEGFA^+^ Neutrophil-Related Signatures; VEGFA: Vascular Endothelial Growth Factor A; ICI: Immune Checkpoint Inhibitors; ROC: Receiver Operating Characteristic; AUC: Area Under the Curve.

## Data Availability

No new data were created or analyzed in this study.

## Abbreviations

The following abbreviations are used in this manuscript:

ROS	Reactive oxygen species
NETs	Neutrophil extracellular traps
AI	Artificial intelligence
ML	Machine learning
DL	Deep learning
HSCs	Hematopoietic stem cells
MZ	Marginal zone
TANs	Tumor-associated neutrophils
TME	Tumor microenvironment
MDSCs	Myeloid-derived suppressor cells
PMN-MDSCs	Polymorphonuclear myeloid-derived suppressor cells
DAMPs	Damage-associated molecular patterns
VEGF-A	Vascular endothelial growth factor A
MMP-9	Metalloproteinase-9
CHIP-seq	Chromatin immunoprecipitation sequencing
JIA	Juvenile idiopathic arthritis
scRNA-seq	Single-cell RNA sequencing
CyTOF	Mass cytometry
iLDNs	Immature low-density neutrophils
SCENIC	Single-cell regulatory network inference and clustering
MIRI	Myocardial ischemia-reperfusion injury
TBI	Traumatic brain injury
ATP	Adenosine triphosphate
PDAC	Pancreatic ductal adenocarcinoma
GBC-LI	Gallbladder cancer liver invasion
SVM	Support vector machines
RF	Random forest
PCA	Principal component analysis
FNNs	Feedforward neural networks
GCNs	Graph convolutional networks
AEs	Autoencoders
GANs	Generative adversarial networks
GPT	Generative pretrained transformers
CIMLR	Cancer Integration via Multikernel Learning
CLCLSA	Contrastive Learning and Self Attention
NMF	Non-negative matrix factorization
GC	Gastric cancer
LC	Long COVID
MI	Myocardial infarction
ICIs	Immune checkpoint inhibitors
XGBoost	Extreme Gradient Boosting
GLM	Generalized linear models
SVM-RFE	Support Vector Machine–Recursive Feature Elimination
AAA	Abdominal aortic aneurysm
CNN	Convolutional neural networks
CTA	Computed tomography angiography
CRC	Colorectal cancer
